# Is there an Intrinsic Relationship between LFP Beta Oscillation Amplitude and Firing Rate of Individual Neurons in Macaque Motor Cortex?

**DOI:** 10.1093/texcom/tgaa017

**Published:** 2020-05-13

**Authors:** Joachim Confais, Nicole Malfait, Thomas Brochier, Alexa Riehle, Bjørg Elisabeth Kilavik

**Affiliations:** 1 Institut de Neurosciences de la Timone (INT), UMR 7289, CNRS, Aix-Marseille Université, Marseille 13005, France; 2 Institute of Neuroscience and Medicine (INM-6), Jülich Research Centre, Jülich 52428, Germany; 3 Cynbiose, Marcy l’Étoile 69280, France

**Keywords:** beta amplitude, local field potential, monkey, motor cortex, spike count

## Abstract

The properties of motor cortical local field potential (LFP) beta oscillations have been extensively studied. Their relationship to the local neuronal spiking activity was also addressed. Yet, whether there is an intrinsic relationship between the amplitude of beta oscillations and the firing rate of individual neurons remains controversial. Some studies suggest a mapping of spike rate onto beta amplitude, while others find no systematic relationship. To help resolve this controversy, we quantified in macaque motor cortex the correlation between beta amplitude and neuronal spike count during visuomotor behavior. First, in an analysis termed “task-related correlation”, single-trial data obtained across all trial epochs were included. These correlations were significant in up to 32% of cases and often strong. However, a trial-shuffling control analysis recombining beta amplitudes and spike counts from different trials revealed these task-related correlations to reflect systematic, yet independent, modulations of the 2 signals with the task. Second, in an analysis termed “trial-by-trial correlation”, only data from fixed trial epochs were included, and correlations were calculated across trials. Trial-by-trial correlations were weak and rarely significant. We conclude that there is no intrinsic relationship between the firing rate of individual neurons and LFP beta oscillation amplitude in macaque motor cortex.

## Introduction

The properties of motor cortical local field potential (LFP) beta oscillations have been the focus of many studies. They occur as bursts (Murthy and Fetz 1996; Donoghue et al. 1998; Feingold et al. 2015), typically lasting 100–500 ms and not locked to external events. The probability of observing beta bursts changes across trial epochs however (e.g., Feingold et al. 2015), leading to the notion of these oscillations being loosely “event-related”. Soon after their first description (Berger 1929), human sensorimotor beta oscillations were linked to states of neuronal activity equilibrium (Jasper and Penfield 1949). Subsequently, beta event-related synchronization (ERS) and desynchronization (ERD) were interpreted as reflecting deactivation and activation, respectively, of the sensorimotor cortex (Pfurtscheller et al. 1996; Salenius et al. 1997; Pfurtscheller and Lopes da Silva 1999; Pfurtscheller 2001; Neuper et al. 2006; Bechtold et al. 2018). This concept mainly springs from the robust observations of much reduced beta oscillation amplitude just before and during movements (Kilavik et al. 2013). However, the notion that motor cortical beta ERD/ERS indexes neuronal activation/deactivation (Neuper et al. 2006) might suggest that there is generally an inverse relationship between neuronal spike rate and beta amplitude.

Several studies examined the relationship between macaque motor cortical LFP beta oscillations and local spiking activity (e.g., Murthy and Fetz 1996; Donoghue et al. 1998; Baker et al. 1999; Denker et al. 2011; Canolty et al. 2012; Engelhard et al. 2013; Best et al. 2017; Rule et al. 2017, 2018; Riehle et al. 2018). Importantly, pioneering studies examined the relationship between LFP beta amplitude and neuronal spike rate (Murthy and Fetz 1996; Donoghue et al. 1998). Murthy and Fetz (1996) found no modulations in the firing rate of neurons in relation to beta amplitude, whereas Donoghue et al. (1998) found some motor cortical locations with increased firing rates during increased oscillation amplitude, and others showing the opposite.

Unfortunately, the discrepancy between these findings remains overlooked and unresolved in recent work. Canolty et al. (2012) reported negative or positive correlations between LFP beta amplitude and the firing rate of a majority of individual neurons, which they termed “amplitude-to-rate” mapping. This mapping could be inversed across different behavioral contexts (manual control vs. brain control task). They therefore proposed that “task-dependent changes in the beta-to-rate mapping play a role in the transient functional reorganization of neural ensembles”. Following up on these results, it was proposed that by means of this amplitude-to-rate mapping, beta activity could mediate switches between neuronal subnetworks (Hutchison et al. 2013; Womelsdorf et al. 2013; see also Ardid and Wang 2013 and Cannon et al. 2014). Importantly, this assumes an intrinsic relationship between beta amplitude and firing rate. If so, noninvasive studies having access only to observations of beta amplitude modulations can aim for insights into the underlying neuronal firing rate dynamics (e.g., Tzagarakis et al. 2015), and compare these across behavioral contexts (Schnitzler et al. 1997; Hari et al. 1998; Kilner et al. 2009; Avanzini et al. 2012; Brinkman et al. 2014). However, Rule et al. (2017) found no consistent relationship between beta amplitude and neuronal firing rate, but did not discuss their results in relation to those of Canolty et al. (2012). Indeed, differences in analysis approaches may explain the divergent conclusions reached in the different studies. Canolty et al. (2012) analyzed data across all trial epochs, whereas Rule et al. (2017) restricted their analyses to steady-state movement preparation periods.

To help resolve this controversial issue, we correlated macaque motor cortical LFP beta oscillation amplitude with neuronal spike counts recorded during visuomotor behavior (Confais et al. 2012; Kilavik et al. 2012). First, in an analysis we termed task-related correlation, single-trial data collected during all trial epochs were included. Thus, the concurrent modulations in the 2 signals, related to the unfolding trial events and related behavior, can be expected to influence the amount of correlation observed between them. Task-related correlations were often significant. However, these correlations remained equally strong in a trial-shuffling control analysis, in which beta amplitude and spike count were randomly recombined from different trials but the same trial epoch. The task-related correlations do not therefore reflect a fine temporal coordination between the 2 signals, but rather concurrent task-related modulations that remain sufficiently similar across trials. Second, in an analysis we termed trial-by-trial correlation, only data from fixed trial epochs were included, and correlations were calculated across trials. Trial-by-trial correlations were weak and rarely significant.

In conclusion, we found no intrinsic relationship between the firing rate of individual neurons and LFP beta oscillation amplitude in macaque motor cortex, beyond each of these signals being systematically, yet independently, modulated by external factors such as the behavioral task. We terminate with a concise overview of the relevant literature and discuss how our results might reconcile the different conclusions therein.

## Materials and Methods

We analyzed LFP signals and spiking data recorded simultaneously on multiple electrodes in the motor cortex of 2 macaque monkeys during the performance of a visuomotor delayed center-out reaching task. We used previously collected data, from which other results have been obtained and presented (Kilavik et al. 2010, 2012, 2014; Ponce-Alvarez et al. 2010; Confais et al. 2012). We have already shown that this dataset contains strong LFP oscillations in the beta range, which are systematically modulated in amplitude and peak frequency by the behavioral task (Kilavik et al. 2012). We have also reported on robust and specific modulations in neuronal spiking activity in relation to the behavioral task (Confais et al. 2012). The experimental data (preprocessed LFPs and sorted spikes as outlined in the Data Selection and Analysis section) can be made available upon request to the authors, to researchers affiliated with a research institution, upon a brief and rational explanation about the intended use of the data.

### Animal Preparation and Data Recording

Two adult male Rhesus monkeys (T and M, both 9 kg) participated in the study. Care and treatment of the animals during all stages of the experiments conformed to the European and French Government Regulations applicable at the time the experiments were performed (86/609/EEC).

After learning an arm-reaching task (see below) the monkeys were prepared for multielectrode recordings in the motor cortex of the right hemisphere, contralateral to the trained arm. The recording chamber positioning above primary motor and dorsal premotor cortices was verified with T1-weighted MRI scans in both monkeys and also with intracortical microstimulation in monkey M (see details in Kilavik et al. 2010). Across all included recording locations, the sampled regions spanned about 4 and 13 mm diameter on the cortical surface in monkeys T and M, respectively (Kilavik et al. 2010), and were in majority arm/hand related.

A multielectrode, computer-controlled microdrive (MT-EPS, AlphaOmega, Nazareth Illith, Israel) was used to transdurally insert up to 4 or 8 (in monkeys T and M, respectively) microelectrodes. The reference was common to all electrodes and positioned, typically together with the ground, on a metal screw on the saline-filled metallic recording chamber. In monkey T the electrodes were always organized in a bundle in one common larger guide tube holding the individual electrode guides, with an interelectrode distance <400 μm (MT; AlphaOmega). However, since each electrode was driven separately, depth varied across electrodes. In monkey M, on some days electrodes were organized in a bundle as for monkey T, and on others the electrodes were positioned independently within the chamber with separate guide tubes (Flex-MT; AlphaOmega), thus resulting in up to 13 mm interelectrode distance. The amplified raw signal (1 Hz–10 kHz) was digitized and stored at 32 kHz. For the online extraction of single neuron activity, the amplified raw signal was hardware high-pass filtered at 300 Hz to obtain the high-frequency signal, on which an online spike shape detection method was applied (MSD, AlphaOmega, Nazareth Illith, Israel), allowing isolation of up to 3 single neurons per electrode. The timing of each spike was then stored as TTLs at a temporal resolution of 32 kHz, down-sampled offline to 1 kHz before analysis. Offline spike sorting on the raw signals was additionally performed in Matlab (The MathWorks Inc., USA) by using Principal Component Analysis in the toolbox MClust (http://www.stat.washington.edu/mclust/) when the online spike sorting was considered as nonoptimal. In parallel, the amplified raw signal was hardware low-pass filtered online at 250 Hz to obtain the low-frequency LFP signal, which was stored with a temporal resolution of 1 kHz. Behavioral data were transmitted online to AlphaMap (AlphaOmega) from the CORTEX software (NIMH, http://dally.nimh.nih.gov), which was used to control the task.

### Behavioral Task

**Figure 1:**
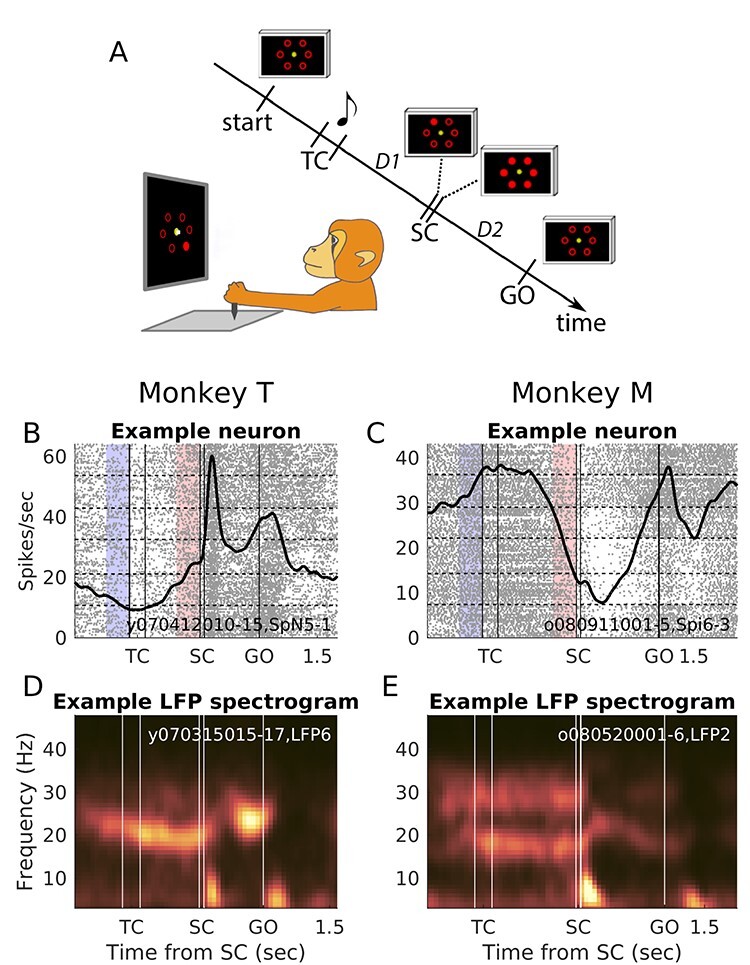
Behavioral paradigm and single neuron and LFP examples. (*A*) Behavioral paradigm. Left, drawing of the experimental apparatus showing the SC epoch (with the hand cursor on the central fixation dot). Right, sequence of task events, not to scale. Start indicates the moment when the monkey brings the hand cursor to the center of the screen to initiate a new trial. The musical note indicates the presentation of a tone. Tone pitch differs according to delay duration. All displays shown in the diagram stay on until the next one appears (hand cursor is not shown). TC, 200 ms; SC, 55 ms; D1, delay 1; D2, delay 2. Both delays have either short duration (700 ms in monkey T and 1000 ms in monkey M) or long duration (1500 ms in monkey T and 2000 ms in monkey M). There is also a 700 ms delay between start and TC. (*B* and *C*) Raster plots and peri-stimulus time histograms (PSTHs) of 2 example neurons, in short delay trials, 1 for each monkey (session and neuron ID indicated inside plots). In the raster plot, each dot is an action potential and each row a trial, ordered vertically according to the 6 target/movement directions. The thick black line represents the neuronal activity averaged across all the shown trials (PSTH; smoothed with a Gaussian filter of length 100 ms and sigma 50 ms). The thin vertical lines mark each task event (TC on/off, SC on/off and GO). The horizontal dashed lines separate the different movement directions (clockwise shift of direction starting with movement toward 3 o’ clock from the bottom to the top of the raster displays). The epochs marked in light red preceding SC and light blue preceding TC were used for the trial-by-trial correlation analyses. (*D* and *E*) Spectrograms of 1 representative example LFP for each monkey, including all correct short delay trials (session and LFP number indicated inside plots). Frequency is on the vertical axis and time along the horizontal axis. Warmer colors indicate increased power (a.u.) using a perceptually flat color-map (Crameri 2018), with color limits set to the minimum and maximum power values above 10 Hz, separately for each monkey. To create the spectrograms, the LFPs were first high-pass filtered at 2 Hz with a fourth order Butterworth filter before the power spectral density (based on discrete Fourier transform) was calculated, at 1 Hz frequency resolution. The averages across all trials were plotted at the center of each sliding window (300 ms duration, 50 ms shifts). The brief power-increases below 10 Hz after SC and GO reflect visual and movement evoked potentials.

We trained the 2 monkeys to make arm-reaching movements in 6 directions in the horizontal plane from a common center position, by holding a handle that was freely movable in the two-dimensional plane (Fig. *1A*). In some sessions, only 2 randomly chosen opposite directions were used to reduce the session duration, concerning 21% and 39% of the analyzed sessions in monkeys T and M, respectively. The monkeys had continuous feedback about hand (white cursor) and the 6 possible target positions (red outlines) on a vertical monitor in front of them.

Two delays were presented successively in each trial. The 2 delays (D1 and D2) had the same fixed duration, either short or long. Thus, in total, there were 12 (or 4) task conditions, combining 6 (or 2) directions and 2 delay durations. The delay duration was instructed by an auditory cue just before D1 initiation, set from trial to trial in a pseudorandom fashion. Durations were either 700 or 1500 ms for monkey T, 1000 or 2000 ms for monkey M. The monkey started each trial by moving the handle to the center (“start” in Fig. 1*A*) and holding it there for 700 ms until a temporal cue (TC) was presented. TC consisted of a 200 ms long tone, its pitch indicating the delay duration (low or high pitch for short or long delay, respectively), starting at its end. The delay following TC (D1) involved temporal attention processes (Confais et al. 2012) to perceive the spatial cue (SC) that was illuminated very briefly (55 ms) at its end at one of the peripheral target positions. To assure the temporal precision of SC illumination time and duration, light-emitting diodes (LEDs) were used, which were mounted in front of the computer screen in fixed positions at the center of the 6 peripheral red target outlines, on a transparent plate. SC was subsequently masked by the additional illumination of the 5 remaining LEDs, marking the start of D2. During D2 the movement direction indicated by the visual cue SC had to be memorized and prepared. All LEDs went off at the end of D2 (GO signal), indicating to the monkey to reach toward and hold (for 300 ms) the correct peripheral target position. In summary, during D1 the monkey had to wait for SC, which was briefly presented at the end of the precued time interval. D2 entailed visuomotor integration and movement preparation while waiting for the GO signal.

The reaction and movement times were computed online to reward the monkey after target hold, with a maximum allowance of 500 ms for each. For data analysis, the reaction times were redefined offline using the arm trajectories. Trajectories were measured in *x* and *y* vectors at 1 ms resolution. The mean of each *x* and *y* vector during the 500 ms before GO in each trial was used as the movement starting position. The moment when reaching a 2 mm position deviation, minus a fixed latency of 35 ms (average movement duration from the starting position to the threshold), was determined as movement onset, separately for the *x* and *y* vectors. From each of the 2 vectors, the shortest time was defined as RT. These values were controlled by visual inspection of single trial trajectories (see Kilavik et al. 2010).

### Data Selection and Analysis

While the monkeys performed the reaching task, we recorded neuronal activity from the motor cortex. We recorded 90 sessions in 37 days in monkey T and 151 sessions in 73 days in monkey M. Consecutive sessions in the same day were made after lowering further the electrodes to sample new neurons. This provided a total of 287 and 759 individual recording sites in monkeys T and M, respectively. A site is defined as the conjunction of a specific chamber coordinate of the electrode entry and the cortical depth. After site elimination due to insufficient recorded trials, or large recording artifacts affecting either the lower (LFP) or higher (spiking activity) frequencies, 123 and 352 sites remained for further analyses, from 65 and 134 individual sessions, for monkeys T and M, respectively. These essentially constitute the conjunction between the LFP datasets studied in Kilavik et al. (2012) and the single neuron datasets studied in Confais et al. (2012).

**Figure 2:**
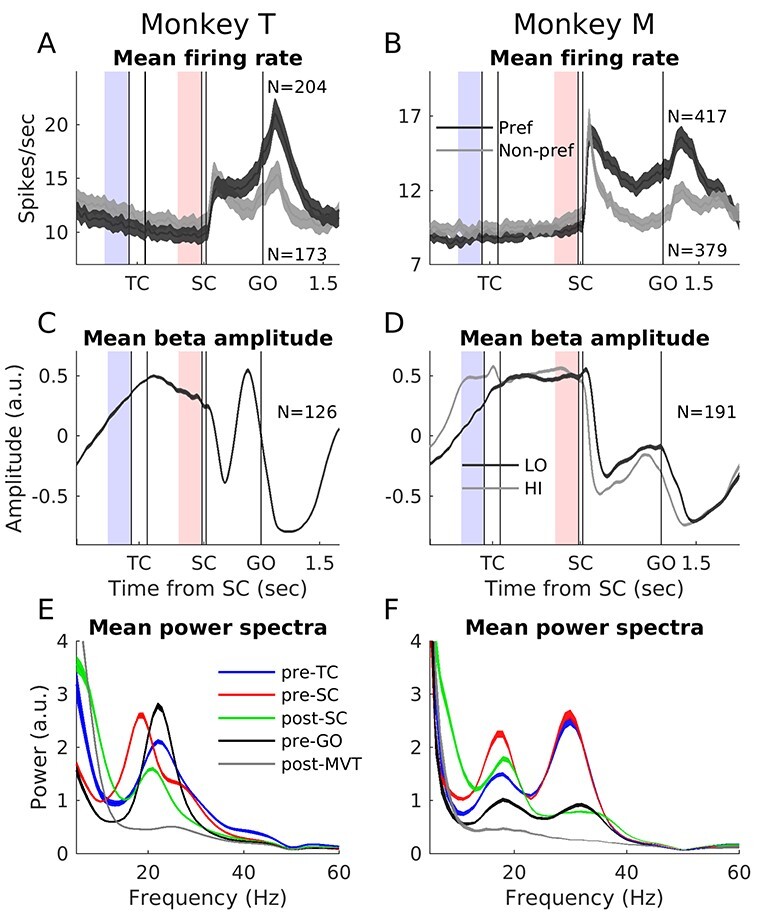
Mean neuronal firing rate, LFP beta amplitude and power spectra. (*A* and *B*) Mean firing rate for all neurons included in the task-related correlation analysis, for preferred (dark gray) and nonpreferred (light gray) movement directions, in short delay trials for monkey T (left) and monkey M (right). The curves reflect the mean firing rate ± standard error of the mean (SEM) across neurons. The mean rate for each SUA was smoothed with a Gaussian filter of length 50 ms and sigma 20 ms, before averaging. *N* indicates the number of included neurons. *N* is smaller in the nonpreferred direction due to the 3 Hz inclusion criterion (see Materials and Methods). This inclusion criterion also causes a slightly higher population firing rate prior to SC for the nonpreferred direction. Per definition the rate is lower after SC for the nonpreferred compared with the preferred direction, and the somewhat fewer neurons for the nonpreferred direction have on average slightly higher rate from the start of the trial. Data between trial start and until 1000 ms after the GO signal (as depicted) were included in the task-related correlation analysis. The epochs marked in light red preceding SC and light blue preceding TC were used for the trial-by-trial correlation analysis. (*C* and *D*) Average LFP beta amplitude for all LFPs. LFPs that were part of multiple beta-neuron pairs were included only once (*N* shown for each monkey). The curves reflect the mean amplitude ± SEM across LFPs. LFP beta amplitude was estimated from the analytical signal (Hilbert transform), as described in the methods. Before averaging across LFPs we normalized the beta amplitude of each LFP by subtracting the mean and dividing by the standard deviation. The epochs in light red preceding SC and light blue preceding TC were used for the trial-by-trial correlation analysis and their power spectra are shown in the red and blue curves in (*E* and *F*), respectively. (*E* and *F*) Average LFP power spectra in 5 different 300 ms duration trial epochs, either aligned to external task events (pre-TC, pre-SC, post-SC, and pre-GO) or to movement initiation (post-MVT). The curves reflect the mean power ±SEM across LFPs, for the same LFPs that are included in (*C*) and (*D*). Before spectral analysis, each LFP was treated offline with a narrow 50-Hz “notch” filter (eighth order Butterworth band-stop filter with stop band 49–51 Hz). The power spectral density (based on discrete Fourier transform at 1 Hz frequency resolution) for each LFP was normalized to the mean power across all epochs between 10 and 40 Hz before averaging across LFPs.

All analyses were conducted offline by using Matlab (The MathWorks, Inc.). We studied a low LFP beta band that was strong in both animals. In addition, in monkey M who also had a marked beta band at higher frequency (see Kilavik et al. 2012 and Figs 1*E* and 2*F*), the analysis was repeated for this band. We first band-pass filtered the LFP around the average peak beta frequency for each band with a zero-phase fourth order Butterworth filter. In monkey T the LFPs were filtered between 22 ± 5 Hz to capture the dominant low beta band across the entire trial (see example in Fig. 1*D* and average power spectra across all LFPs in Fig. 2*E*). For monkey M, to capture the low and high beta bands across the entire trial, the LFPs were filtered at 19 ± 5 Hz and 32 ± 5 Hz, respectively (see Figs 1*E* and 2*F*). After filtering, beta oscillation amplitude was estimated from the analytical filtered LFP, as the envelope of the signal from the Hilbert transform.

From the online and offline spike sorting, typically 1–3 neurons were available on each electrode. For the correlation analyses between LFP beta amplitude and neuronal firing rate, beta-neuron pairs were constructed using signals from different simultaneously recorded sites. This choice was guided by findings demonstrating the possibility of spike contamination in the LFP signal recorded on the same electrode, including the lower LFP frequency ranges studied here (Zanos et al. 2011; Waldert et al. 2013). From the 127 and 358 included sites, 314 and 661 beta-neuron pairs were constructed in monkeys T and M, respectively. Each neuron was paired with only one LFP. As described in the data recording details above, we used 2 different electrode microdrives with different interelectrode spacing. For all pairs in monkey T, and 60% (398/661) of pairs in monkey M, the corecorded site used for LFP beta was less than 400 μm away from the neuron site in chamber coordinates (but at different cortical depths). The remaining 40% (263/661) of pairs in monkey M were recorded with the microdrive using independently positioned guide tubes. Still, only 16% of all beta-neuron pairs in this animal (106/661) combined sites more than 2 mm apart, and only 1% (7/661) more than 5 mm apart. Whenever multiple corecorded sites were available, the site selection for LFP beta was mainly driven by LFP signal quality. Different LFPs recorded several millimeters apart in motor cortex typically show very similar modulations in beta amplitude (Kilavik et al. 2012; Denker et al. 2018). In monkey M we found no significant difference in the correlations (described below) between the subsets of pairs recorded with the 2 different microdrives. However, as a control, we also replicated the correlation analyses in pairs combining neuron and LFP from the same electrode. This control yielded an identical pattern of results (not presented) as obtained when combining signals from different electrodes (described below). In particular, the proportions of pairs with significant task-related and trial-by-trial correlations, the proportions of positive and negative correlations, and the broadness of the distributions of correlation coefficients remained the same for the 2 ways of combining signals into pairs.

In the constructed beta-neuron pairs, some trials with obvious artifacts (mainly due to teeth grinding or static electricity) detected by visual inspection, were excluded from further analysis (less than 5% of all trials). After trial elimination, and considering the variable duration for which the monkeys were willing to work in different behavioral sessions, the analyzed beta-neuron pairs contained at least 8 correct trials in each condition, although typically 20 or more correct trials were available per condition. The average number of correct trials in each condition across pairs was 23 ± 5 (mean ± standard deviation) for monkey T and 20 ± 5 for monkey M. The average number of total short (long) delay trials for each pair was 117 ± 36 (117 ± 37) for monkey T and 93 ± 36 (90 ± 36) for monkey M.

### Task-Related Correlations between LFP Beta Amplitude and Neuronal Spike Count

We termed task-related correlation the correlation between 2 brain signals calculated across different trial epochs, such that the concurrent, yet independent, modulations in the brain signals related to the unfolding task events and related behavior can be expected to influence the amount of correlation observed between them. The task-related correlation was calculated between LFP beta oscillation amplitude and neuronal spike count for each beta-neuron pair. Data recorded in all epochs between the trial start (initial central touch) and until 1000 ms after the GO signal were included (as displayed in all figures), analyzed separately for short and long delay trials. Across the included sessions, the average reaction time in short (long) delay trials was 161 ± 35 (206 ± 26) ms in monkey T and 232 ± 45 (255 ± 26) ms in monkey M, and the average movement time was 302 ± 19 (295 ± 20) ms in monkey T and 298 ± 26 (303 ± 28) ms in monkey M (see Kilavik et al. 2010). Thus, average reaction and movement times were both shorter than their maximally allowed durations of 500 ms each, so that the analysis typically also included most of the required 300 ms target-hold time.

The beta-neuron correlations were calculated separately for the preferred and nonpreferred (opposite) movement directions for the neuron in each pair, where preferred direction was taken as the one with maximal trial-averaged spike rate any time after the presentation of SC up to trial end. This was done to evaluate whether the task-related beta-neuron correlation depended on the involvement of the neuron in coding for the cued movement.

**Figure 3:**
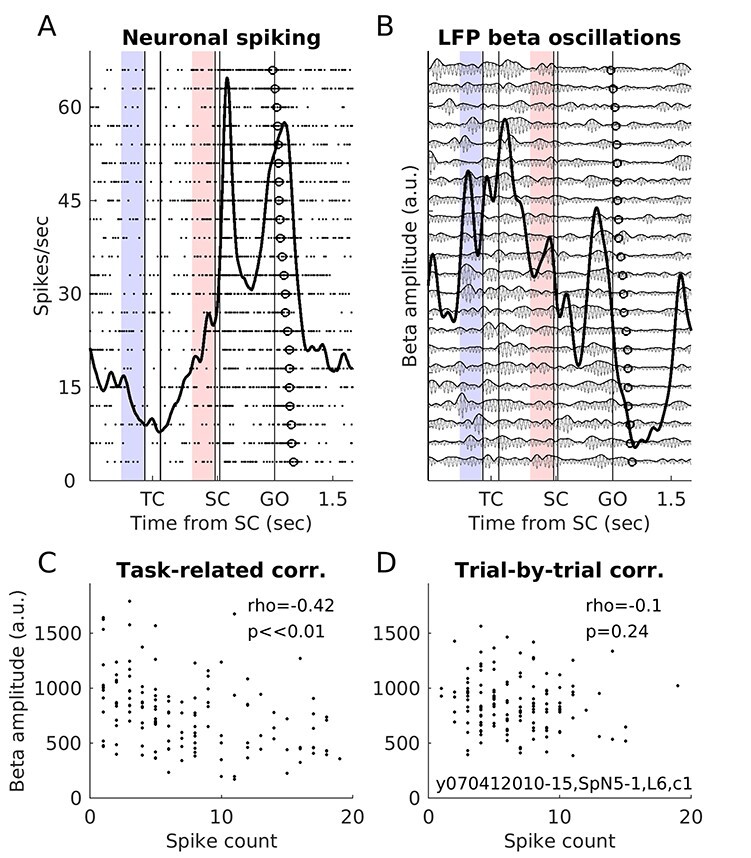
Example beta-neuron pair. (*A*) Raster plot and PSTH of 1 example neuron in its preferred movement direction, in short delay trials (from monkey T; session, neuron and LFP ID, and condition indicated inside plot D; same neuron as in Fig. 1*B*). In the raster plot, each dot is an action potential and each row a trial, ordered vertically according to reaction times (open circles; shortest on top). The thick black line represents the neuronal activity averaged across all the shown trials (PSTH; smoothed with a Gaussian filter of length 100 ms and sigma 50 ms). The epoch marked in light red preceding SC (also in *B*) was used for the pre-SC trial-by-trial correlation analysis shown in *D* (that also included the short delay trials for all the other movement directions). The light blue epoch preceding TC (also in *B*) was used for the pre-TC trial-by-trial correlation analysis (not shown). (*B*) LFP from another co-recorded electrode, filtered for the beta range (22 ± 5 Hz; light gray curves), for the same trials as for the neuron in *A*. Darker gray curves show the instantaneous beta oscillation amplitude, which was estimated from the analytical filtered LFP as the Hilbert transform signal envelope. The thick black line indicates the average beta amplitude across all shown trials (smoothed with a Gaussian filter of length 100 ms and sigma 50 ms). (*C*) This pair’s task-related correlation, for short delay trials in the preferred direction. Each dot corresponds to one 300 ms window, with combined single values of mean beta amplitude and spike count (*N* = 135). The Spearman’s rho was −0.42, a highly significant negative correlation. (*D*) This pair’s trial-by-trial correlation in the pre-SC epoch, for short delay trials (*N* = 135). Each dot corresponds to the mean beta amplitude and spike count for 1 trial, in the 300 ms pre-SC window marked in light red in (*A*) and (*B*). The correlation was not significant. The pre-TC trial-by-trial correlation in this pair was also not significant (not shown).

The single trial data in these 2 directions were cut in 300 ms nonoverlapping consecutive windows. The window duration of 300 ms was firstly chosen based on the typical duration of beta bursts in our dataset (200–500 ms), see example in Figure 3*B*; see also Murthy and Fetz (1996). Note that recent literature suggests that in some contexts, beta bursts can be of much shorter duration than seen in our dataset (e.g., Feingold et al. 2015; Lundqvist et al. 2016; Sherman et al. 2016). Since these 300 ms windows were aligned to the task timing, for example, signal occurrences, and beta bursts do not have a fixed temporal relationship with such external events, some windows will overlap with a beta burst, while others will fall in a period with low beta amplitude, and some will partly overlap with a beta burst.

Secondly, the window duration of 300 ms was considered to be the minimal duration needed for meaningful (nonzero) spike counts in a majority of individual windows. However, we additionally restricted our analysis to the subsets of beta-neuron pairs for which the average firing rate of the neuron, across all 300 ms windows, was above 3 Hz. The numbers of analyzed pairs thus varied slightly for short and long delay trials and for preferred and nonpreferred movement directions, as detailed in Table 1 (see also Fig. 2*A*,*B*).

**Table 1:** Summary of results from correlation analyses^a^

Analysis	Data selection	Monkey T			Monkey M LO			Monkey M HI		
		Significant correlations	Negative correlations	Stronger correlation than trial-shuffles	Significant correlations	Negative correlations	Stronger correlation than trial-shuffles	Significant correlations	Negative correlations	Stronger correlation than trial-shuffles
Task-related	Short pref.	65/204 (32%)	57/65 (88%)	4/65 (2%)	75/417 (18%)	61/75 (81%)	7/75 (2%)	113/417 (27%)	93/113 (82%)	11/113 (3%)
	Short nonpref.	43/173 (25%)	26/43 (60%)	0/43	60/379 (16%)	37/60 (62%)	4/60 (1%)	82/379 (22%)	48/82 (59%)	9/82 (2%)
	Long pref.	55/194 (28%)	44/55 (80%)	3/55 (2%)	62/408 (15%)	50/62 (81%)	12/62 (3%)	76/408 (19%)	70/76 (92%)	11/76 (3%)
	Long nonpref.	34/180 (19%)	18/34 (53%)	1/34 (1%)	44/371 (12%)	22/44 (50%)	7/44 (2%)	59/371 (16%)	34/59 (58%)	9/59 (2%)
Trial-by-trial pre-SC	Short	7/173 (4.0%)	3/7		21/369 (5.7%)	5/21		17/369 (4.6%)	8/17	
	Long	6/171 (3.5%)	4/6		18/373 (4.8%)	9/18		13/373 (3.5%)	6/13	
Trial-by-trial pre-TC	Short	2/171 (1.2%)	2/2		25/340 (7.4%)	12/25		28/340 (8.2%)	15/28	
	Long	6/170 (3.5%)	5/6		20/349 (5.7%)	7/20		25/349 (7.2%)	8/25	

^a^Numbers and percentages of beta-neuron pairs with significant task-related and trial-by-trial correlations are presented separately for Monkey T and monkey M low (LO) and high (HI) beta bands. Short and long delay trials are presented separately. Neuronal preferred and nonpreferred movement directions are presented separately for task-related correlations. The proportions of significant correlations with negative sign are specified in separate columns. For the task-related correlation analyses, the numbers of pairs with a significantly stronger correlation coefficient in the original data than in the trial-shuffled resampling control data are specified in separate columns, and their percentages are shown with respect to the total number of analyzed pairs.

This trial cutting provided 11 (16) nonoverlapping 300 ms windows in monkey T and 13 (19) in monkey M, for short (long) delay trials. The total number of windows accumulated across trials varied because of variable number of correctly performed trials across sessions. The average number of overall available windows for all trials in the same (preferred or nonpreferred) movement direction in short (long) delay trials was 259 ± 64 (373 ± 96) for monkey T and 283 ± 77 (400 ± 113) for monkey M. There were typically more than twice as many windows available for this task-related correlation analysis compared with the number of trials available for the trial-by-trial correlation analysis that will be described in the next section (averages of 117 trials in both short and long for monkey T and 93 and 90 trials in short and long, respectively, for monkey M; see above). This difference may pose problems in comparing the task-related and trial-by-trial correlation effects, due to sample size affecting the statistical power. To permit a fair comparison, the task-related correlation was therefore quantified for each beta-neuron pair after first selecting from the total available windows a subset equaling the number of short (or long) delay trials for that pair. This selection was in a first step done such that every second window was excluded. The exclusion of every second window was then repeated if there were still too many remaining windows. Finally, this selection was complemented with reintroduced windows if needed (randomly selected from the previously excluded), to arrive at the correct number of windows.

**Figure 4:**
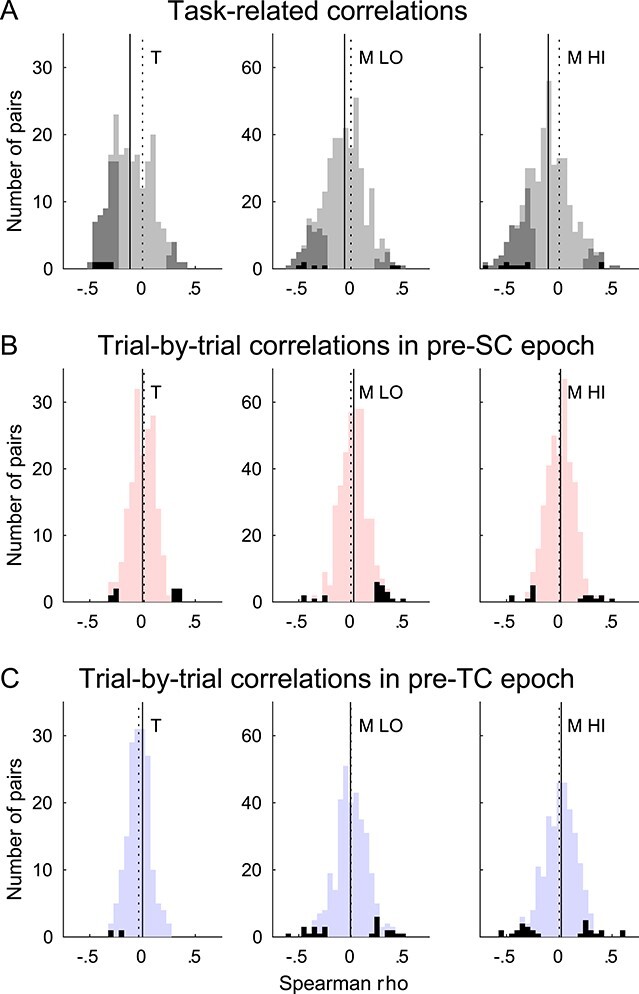
Task-related and trial-by-trial correlations. (*A*) Complete distributions of Spearman’s rho values for task-related correlations in neuronal preferred movement direction in short delay trials for monkey T (left) and monkey M low beta band (LO; middle) and high beta band (HI; right). All beta-neuron pairs are shown in light gray. Overlaid in dark gray are the significant pairs (*P* < 0.01). Overlaid in black are those significant pairs that in addition had a significantly stronger correlation in the original data than in their trial-shuffled controls. Dotted vertical lines mark zero (also in *B* and *C*). Solid vertical lines mark the medians of the complete (light gray) distributions (also in *B* and *C*), which were significantly shifted to the left (negative correlations; Wilcoxon signed rank test on Fisher’s z-transformed rho values; *P* << 0.01 for all datasets). (*B*) Distributions of trial-by-trial correlations in short delay trials in the pre-SC epoch. All pairs are shown in light red. Overlaid in black are the significant pairs (*P* < 0.01). The complete distributions were centered on zero for monkey T (*P* = 0.58) and monkey M for the high band (*P* = 0.28) and only slightly shifted to the right (positive correlations, *P* < 0.01) for monkey M for the low band. (*C*) Distributions of trial-by-trial correlations in short delay trials in the pre-TC epoch. All pairs are shown in light blue. Overlaid in black are the significant pairs (*P* < 0.01). The complete distributions were centered on zero for monkey M low and high bands (*P* = 0.91 and *P* = 0.14, respectively) and slightly shifted to the left (negative correlations, *P* < 0.01) for monkey T.

The average beta amplitude (Hilbert envelope; see Fig. 3*B*) and the spike count in each selected 300 ms window (providing 1 value per signal type per window) were then used to calculate the beta-neuron task-related correlation, quantified with the Spearman’s rank order correlation (Spearman’s rho; see example in Fig. 3*C* and complete results in Fig. 4 and Table 1). Correlations with *P* < 0.01 were considered significant, but the complete distributions of rho values across the populations of beta-neuron pairs are presented (Fig. 4), to allow appreciating the magnitude of the different types of correlations.

To estimate how often the task-related correlation between beta amplitude and neuronal spike count could be explained by both signals being systematically, yet independently, modulated in relation to the unfolding behavioral task, rather than by any direct and intrinsic relationship between them, we applied a trial-shuffling control analysis. For each beta-neuron pair with significant task-related correlation, we randomly shuffled the trials from which LFP beta amplitude was computed. The aim of such trial-shuffling was to destroy the fine temporal relationship between the 2 signals, while preserving global task-related modulations, common to different trials. For those pairs that remain equally correlated in the trial-shuffled control analysis, one can assume that the correlation stems from concurrent task-related modulations. After shuffling the trials for the beta amplitude, the correlation analysis was performed as for the original data, as described above. This was repeated 1000 times, each time with a different trial-shuffling. If the original data yielded a larger Spearman’s rho value than 990/1000 (equivalent to *P* < 0.01) of the trial-shuffled analyses, the pair was considered to be correlated beyond what can be explained by concurrent, yet independent, modulations by the task.

### Trial-by-Trial Correlations between LFP Beta Amplitude and Neuronal Spike Count

For each beta-neuron pair the trial-by-trial correlation between LFP beta oscillation amplitude and neuronal spike count was calculated in a 300 ms epoch immediately preceding SC (and TC; see below), across all trials, separately for short and long delay trials (light red epoch in Figs 1–3). While the 300 ms window was aligned to SC, beta bursts do not have a fixed temporal relationship with external events (see introduction, and example in Fig. 3*B*). Thus, as explained above for the task-related correlation analysis, in some trials, the window overlapped with a beta burst, in others it coincided with lower beta amplitude, yet in others it partly overlapped with a beta burst.

We chose the pre-SC trial epoch since we considered it to be the epoch in which the monkey’s behavioral state was most likely to be similar across all trials for the same delay duration. During this epoch, the monkey maintained a stable arm position on the central target and was awaiting the presentation of a visual cue. It started between 1.3 and 2.6 s after the monkey had moved his hand cursor into the central target to start a new trial, and 0.4–1.7 s after the end of the presentation of the auditory temporal cue (TC) providing information about delay duration. Furthermore, the movement direction was still unknown, so all directions can be grouped in the analysis, while analyzing short and long delay trials separately. Significant trial-by-trial beta-neuron correlations in the pre-SC epoch may be mainly related to modulations of internal (anticipatory) processes, thereby best reflecting any intrinsic beta-spike relationship, independent of external factors related to the task such as the processing of external visual or auditory sensory cues or overt movements. As for the task-related correlation analysis, the analysis included only the subsets of beta-neuron pairs for which the average firing rate of the neuron in the pre-SC epoch was above 3 Hz. The LFPs were filtered to capture the main beta frequency band(s) for each animal as described above. The average beta amplitude (Hilbert envelope) and the spike count in each trial in this 300 ms epoch were then extracted for analysis (providing one value per signal type per trial window). The trial-by-trial correlation between beta amplitude and spike count for each beta-neuron pair was then quantified as the Spearman’s rank order correlation, as described above for the task-related correlation analysis (see example in Fig. 3*D*; results across all datasets in Table 1 and Fig. 4*B*).

We repeated this trial-by-trial correlation analysis in the 300 ms window immediately preceding TC (light blue epoch in Figs 1–3), even if in this early trial epoch the animal’s behavioral state might have been somewhat less stable across trials than in the pre-SC epoch, notably with residual arm stabilizing movements within the central target. To have comparable statistical power as for the analyses described above, correlations were quantified separately for short and long delay trials, even though in this trial epoch the monkey was still unaware of the delay duration. Results of this analysis across all datasets are reported in Table 1 and Figure 4*C*.

### Variability in Spike Count and Beta Amplitude

To determine to which degree the correlation analyses results were dependent upon the level of individual signal variability, we estimated the variability of the spike count and beta amplitude across analyses windows. This was done for each beta-neuron pair and for data entering the task-related correlation in neuronal preferred direction and for the trial-by-trial correlation in the pre-SC epoch, separately for short and long delay trials. We calculated the coefficient of variation (CV; standard deviation divided by the mean). The CV of each type of signal was then correlated with the Spearman’s rho values of the beta amplitude—neuronal spike count correlations across pairs.

### Phase-Locking of Neuronal Spiking to LFP Beta Phase

To confirm that the LFP beta oscillations were at least partially of local origin, we verified that a substantial proportion of the neurons significantly locked their spiking activity to the LFP beta phase. The proportion of neurons with a significant phase-locking to beta oscillations was quantified using the whole delay D1 (see Fig. 1*A*), for long delay trials. We focused on D1, since beta amplitude was generally strong in both animals, and in both beta bands for monkey M, in this delay. Furthermore, the pre-SC trial-by-trial correlation analysis was performed using the final 300 ms of this delay. We did not attempt any detailed study of potential modulations in locking across trial epochs, as this would go beyond the scope of the current study and this specific control analysis.

To ensure a reliable statistical analysis, only neurons with more than 50 spikes in this delay, accumulated across all trials, were included. The analysis was thus restricted to a subset of 289/314 pairs in monkey T and of 603/661 pairs in monkey *M. beta* phase was extracted from the Hilbert transformation of the beta-filtered LFP, and the phase at each spike time was determined.

To quantify the phase locking, we first used Rayleigh’s test of nonuniformity of circular data (CircStat Matlab toolbox; Berens 2009). To determine whether the locking was significant for individual neurons, a trial-shuffling method was used. Beta oscillations are typically not phase-reset by external events, such that any phase-resetting effects should have minimal effect. This makes trial-shuffling an efficient method for obtaining a “baseline” measure of phase locking, destroying the fine temporal relationship between the 2 signals, while preserving their individual properties such as rhythmicity.

In the trial-shuffling analysis, 1000 repetitions of the phase-locking analysis (Rayleigh’s test) were done while randomly combining beta phases and spike times from different trials. If the original data yielded a z-statistic value from the Rayleigh’s test larger than 990/1000 (equivalent to *P* < 0.01) of the trial-shuffled analyses, the phase-locking of the neuron was considered to be significant.

## Results

The aim of this study was to determine to which degree there is an intrinsic relationship between the amplitude of LFP beta oscillations and firing rate (spike count) of individual neurons in the motor cortex. We correlated motor cortical LFP beta amplitude and neuronal spike count measured in short windows either along the trial including several different trial epochs (task-related correlation) or within fixed trial epochs, but across trials (trial-by-trial correlation). We start with a general description of the typical task-related modulations in firing rate of the included neurons, as well as of the typical task-related modulation of LFP beta amplitude.

Some preliminary results were presented in abstract form (Kilavik and Riehle 2015).

### Modulations in Neuronal Firing Rate and LFP Beta Amplitude during Task Performance

The monkeys performed a visuomotor arm-reaching task (Fig. 1*A*), while we recorded neuronal activity from motor cortex. Figure 2*A*,*B* shows the average firing rates of all neurons included in this study, separated for neuronal preferred and nonpreferred movement direction. At the population level there was a phasic increase in firing rate for both the preferred and nonpreferred directions following the spatial cue (SC). The population firing rate then decreased during the preparatory delay between SC and GO but remained above the pre-SC level in particular for the preferred direction, before increasing again toward and during movement execution after GO. However, as can be seen in the single neuron examples in Figure 1*B*,*C*, the trial-related modulations in activity of individual neurons could be very diverse. Some neurons increased their activity in anticipation of and response to the presentation of the visual spatial cue (e.g., Fig. 1*B*). Others decreased their activity in anticipation of and response to the cue and were more active during movement execution (e.g., Fig. 1*C*).

Example LFP spectrograms for each monkey are shown in Figure 1*D*,*E*. These examples are representative when it comes to the typical beta power and frequency across trial epochs in these datasets, as we already described in detail in Kilavik et al. (2012). Figure 2*C*,*D* show the average beta amplitude across all included LFPs. Notably, monkey T had one dominant beta band, which varied in peak frequency between 19 and 25 Hz (Fig. 2*E*) across trial epochs. Monkey M had 2 dominant beta bands, a low band with varying peak frequency between 17 and 21 Hz and a high band with varying peak frequency between 29 and 34 Hz (Fig. 2*F*). For both monkeys and both bands, beta amplitude decreased after SC and during the movement execution after GO (Fig. 2*C*,*D*). Note that even if these trial-averaged results suggest a prolonged increase in beta amplitude during the delays, as can be seen in the example LFP in Figure 3*B*, in reality, beta oscillations occur in individual bursts of different duration, amplitude and exact timing across trials (see also Feingold et al. 2015; Lundqvist et al. 2016; Sherman et al. 2016).

### Task-Related Correlations between LFP Beta Amplitude and Neuronal Spike Count are Common

We calculated task-related correlations between LFP beta oscillation amplitude and neuronal spike count along trials including different trial epochs, separately for neuronal preferred and nonpreferred movement directions. In order to have comparable statistical power as for the trial-by-trial correlation analysis (see below), the task-related correlation analysis was performed by selecting only as many windows as there were available trials for the trial-by-trial correlation analysis for each individual pair (see Materials and Methods). An example pair with significant task-related correlation in the neuronal preferred direction is shown in Figure 3. This particular pair had a negative task-related correlation between beta amplitude and neuronal firing rate (Fig. 3*C*).

The overall percentages of pairs with significant correlations, for both monkeys and both beta bands, in short and long delay trials and in the neuronal preferred and nonpreferred movement directions are summarized in Table 1. Task-related correlations for neuronal preferred direction were statistically significant (*P* < 0.01) in 15–32% of pairs, across both monkeys and bands, short and long delay trials. Furthermore, the complete distributions of Spearman’s rho values (Fig. 4*A*), including significant and nonsignificant pairs, were rather broad and significantly shifted toward negative values (Wilcoxon signed rank test on Fisher’s z-transformed rho values; *P* << 0.01).

Indeed, even if a combination of negative and positive significant correlations was observed, the large majority of the significant correlations for neuronal preferred directions were negative (80–92% across both monkeys and bands, short and long delay trials). This dominance of negative correlations is possibly due to the systematic decreases in beta amplitude following the visual spatial cue (SC) and during movement execution (see Fig. 2*C*,*D*), which occurs more or less concurrently with phasic increases in firing rate in a majority of neurons in their preferred direction (see Fig. 2*A*,*B*).

In order to evaluate whether the task-related beta-neuron correlation depended on the involvement of the neuron in coding for the cued movement, we also analyzed neuronal nonpreferred movement direction. Here, 12–25% of pairs had significant task-related correlations, across both monkeys and both bands, short and long delay trials (Table 1). However, the proportions of significant negative correlations were smaller than for the preferred direction (50–63% across both monkeys and bands, short and long delay trials). Furthermore, the complete distributions of Spearman’s rho values were only significantly shifted away from zero for monkey M (distributions not shown)**.** When considering the subsets of pairs with significant correlations in both the preferred and nonpreferred directions in short (long) delay trials, hardly any changed correlation sign 0/19 (1/19) in monkey T, 0/33 (0/19) and 2/44 (0/28) in monkey M low and high bands, respectively. Thus, the different proportions of significant negative correlations for preferred vs. nonpreferred directions mainly stem from those pairs being significantly correlated in only 1 of the 2 directions. Finally, the proportions of significant negative and positive correlations were similar for short and long delay trials for the same movement direction.

### The Task-Related Correlations Reflect Independent Modulations with the Task

We wanted to estimate the degree to which the task-related correlation could be explained by both signals being independently modulated in relation to the unfolding behavioral task, by randomly shuffling the trials used to extract LFP beta amplitude. The aim of the trial-shuffling was to destroy any putative direct (intrinsic) relationship between the 2 signals at a fine temporal scale within the trial, while preserving their overall task-related modulations, which we assumed to be similar across trials.

The outcome of this trial-shuffling control is reported in Table 1 and in Figure 4*A*. Only very few pairs had a significantly stronger correlation coefficient in the original analysis compared with their trial-shuffled controls, corresponding to 0–3% of all pairs entering the task-related correlation analyses. This is a strong argument in favor of concurrent, but largely independent, task-related modulations of beta amplitude and neuronal spike count.

### Trial-by-Trial Correlations between LFP Beta Amplitude and Neuronal Spike Count are Rare

Figure 3*D* shows that in the selected example beta-neuron pair, LFP beta amplitude and neuronal spike count did not correlate trial-by-trial in the pre-SC epoch. This was indeed representative of the populations. Only 3.5–5.7% of the pairs had a significant trial-by-trial correlation in this epoch (across both monkeys and both bands, short and long delay trials), with similar proportions of negative and positive correlations (see Table 1). Figure 4*B* shows the distributions of Spearman’s rho values for the pre-SC trial-by-trial correlation analysis in short delay trials for the 3 datasets. The distributions were narrower than for the task-related correlations and only significantly shifted away from zero (positive shift) for the low beta band in Monkey M in short delay trials and for no dataset in long delay trials (not shown).

As a control, we also quantified the trial-by-trial correlations in the pre-TC epoch. As can be seen in the examples in Figure 1*B*,*C*, many neurons had very different firing rates when comparing the pre-TC (light blue) and pre-SC (light red) epochs. Furthermore, beta amplitude and peak frequency also differed between these 2 epochs (Fig. 2). We therefore selected TC as an appropriate control epoch for the trial-by-trial correlation analysis. As in the pre-SC epoch, only few pairs had a significant correlation (1.0–8.2%; see Fig. 4*C* and Table 1). Furthermore, the distributions of all rho values were narrow, and only significantly shifted away from zero (negative shift) for monkey T, in short delay trials.

### No Influence of Signal Variability on Beta-Neuron Correlations

In order to estimate to which degree significant task-related or trial-by-trial correlations were associated with neuron pairs having large individual signal variability, we quantified the variability (CV) of neuronal spike count and beta amplitude across analysis windows.

For spike count, the population distributions of CV magnitude were slightly smaller (two-sample *t*-tests; *P* < 0.01) across the windows in the pre-SC epoch entering the trial-by-trial correlation than across the task-related windows, except for monkey M for short delay trials (*P* = 0.012). However, across beta-neuron pairs, spike-count CV correlated neither with the strength of task-related nor of trial-by-trial correlation (Spearman’s rank order correlation; *P* > 0.01 for all comparisons). In other words, those neurons with large spike count variability were not more likely to have a significant correlation with beta amplitude.

Beta amplitude was much less variable across trials in the pre-SC epoch than across windows included in the task-related correlation analysis (two-sample *t*-tests; *P* << 0.01 for all comparisons). This was to be expected due to the large differences in beta amplitude particularly when comparing delays with the postcue and movement epochs (see Fig. 2*C*,*D*). Still, as can be appreciated in the example in Figure 3*D*, the beta amplitude could still triple on some trials compared with others in the pre-SC epoch. In the same way as for spike count CV, beta amplitude CV correlated neither with beta-neuron trial-by-trial nor task-related correlation strength (Spearman’s rank order correlation; *P* > 0.01). The only significant association was for an increased beta amplitude variability for pairs with higher task-related correlations in long delay trials for the low beta band in monkey M (*P* = 0.006).

### Neurons Lock their Spikes to LFP Beta Oscillation Phase

The LFP is prone to containing a combination of signals generated by local and distant sources (e.g., Kajikawa and Schroeder 2011). To study the relationship between LFP beta oscillation amplitude and local spiking activity, it is essential to verify the likewise local origin of beta oscillations. A significant phase-locking of the spiking activity of the local neuronal population reveals locking of the neurons to synchronized synaptic inputs (of local or distant origin), in turn leading to local postsynaptic currents that contribute to generating the LFP (Pesaran et al. 2018). As a control analysis, we therefore confirmed that the spiking activity of a substantial proportion of the neurons locked to the phase of the LFP beta oscillations. This control was specifically performed in delay D1, of long delay trials, when beta oscillations were prominent. Notably, D1 included the pre-SC epoch within which only very few neurons were found to have a trial-by-trial spike count modulation in relation to beta amplitude, as described in the previous section.

In monkey T, 63.0% of the neurons locked significantly their spikes to the LFP beta phase. In Monkey M, 29.4% and 30.8% of the neurons locked significantly to the low and high beta bands, respectively. Furthermore, only 14.4% of the neurons locked significantly their spikes to both the low and the high bands, such that overall 45.8% of the neurons locked to either the low, high or both LFP beta bands in monkey M. These results therefore suggest that the LFP beta bands in this study were at least partly locally generated. Importantly, there was no systematic difference in locking prevalence of the few neurons with, compared with the many without, a significant trial-by-trial correlation of spike count with beta amplitude in the pre-SC trial epoch.

## Discussion

To reconcile contradictory literature findings concerning the relationship between beta amplitude and neuronal firing rate, we performed systematic quantifications of correlations between macaque motor cortical LFP beta amplitude and spike count of individual neurons during a visuomotor task, in 2 different manners. First, in the analysis called task-related correlation, data obtained across all behavioral trial epochs were included. Such task-related correlations were frequently significant, however equally strong when data were trial-shuffled. Second, in the analysis called trial-by-trial correlation, only data from a fixed trial epoch were included. Trial-by-trial correlations were weak and rarely significant. We conclude that there is no intrinsic relationship between neuronal spike count and beta amplitude, beyond both types of signals being concurrently, yet independently, modulated by external factors such as the behavioral task.

### Disparate Literature Evidence for an Intrinsic Relationship between Motor Cortical Beta Amplitude and Neuronal Firing Rate

The question of whether modulations in beta amplitude are related to modulations in the activation level of local neurons was already examined more than 20 years ago. In a behavioral context in which macaques made reaching movements to a Klüver board, Murthy and Fetz (1996) found no difference in average firing rate of individual neurons inside and outside beta bursts (20–40 Hz) in motor cortex. However, they found a decrease in the variability of firing rate of individual neurons during and just after burst events, compared with just before bursts. They also noted that many neurons were phase-locked to the high-amplitude beta oscillations, which they suggested might be the main reason for the within-burst decreased firing rate variability. Donoghue et al. (1998) analyzed LFPs and neuronal discharge (individual neurons and multiunits) during tasks involving finger or arm movements. One group of multiunits “overlapped” with LFP beta-gamma oscillations (20–60 Hz), increasing their discharge in epochs of increased oscillation amplitude. Another, “mixed” group mainly decreased their discharge during increased beta oscillation amplitude, but also showed some “overlap”. They noted that the consistent patterns for each recorded cortical site suggested a mechanistic link between LFP oscillation amplitude and neuronal rate.

These 2 rather contradictory studies (Murthy and Fetz 1996; Donoghue et al. 1998) cannot be directly compared, since their methods present critical differences (using the spiking activity of single or multi units; considering different LFP frequency ranges; differences in behavioral tasks). More recently, Canolty et al. (2012) described an “amplitude-to-rate mapping” between many individual neurons and beta oscillations. Some neurons exhibited a strong negative correlation and others a strong positive correlation with beta amplitude, and this mapping could change across tasks (manual control or brain control). The notion of an amplitude-to-rate mapping implies an intrinsic relationship between beta amplitude and firing rate, and might be interpreted such that beta activity indexes switches between subnetworks across different trial epochs, and different tasks (Hutchison et al. 2013; Womelsdorf et al. 2013). The impact of movement initiation upon such beta-neuron correlations was recently demonstrated by Khanna and Carmena (2017), who only analyzed beta amplitude and neuronal firing rate in the epoch surrounding movement onset, confirming the findings of Canolty et al. (2012). Furthermore, using trial-averaged analyses, Spinks et al. (2008) demonstrated a negative correlation between the selectivity for different grasp types in the mean neuronal firing rates and the mean M1 beta power during object holding.

Rule et al. (2017) also addressed the same question, finding no consistent relationship between beta amplitude and spike rate when restricting their analysis to steady-state precue and premovement periods. Noteworthy, Engelhard et al. (2013) trained macaques to increase motor cortical 30–40 Hz LFP oscillation power and spike synchrony. They found no systematic modulation in neuronal firing rate when comparing periods with low and high LFP power.

### Reconciling these Findings—No Intrinsic Relationship beyond Comodulations Driven by Task Events

A comparison of the recent studies by Canolty et al. (2012) and Rule et al. (2017) suggests that their discrepant conclusions might be due to different analysis approaches, either including data from all trial epochs or restricted to steady-state periods, respectively. The 2 ways in which the data were analyzed in our study, quantifying both task-related and trial-by-trial correlations in the same dataset, therefore help resolve this controversy. We found task-related correlations to be common, but for most pairs equally strong when trials were shuffled. Thus, independent task-related modulations of beta amplitude and neuronal spike count that were systematic across trials were sufficient to account for the task-related correlations. The largely absent trial-by-trial correlations within restricted trial epochs confirmed the lack of any intrinsic relationship between beta amplitude and spike count of individual neurons.

One should note that Rule et al. (2017) compared firing rate inside and outside of beta bursts across a 1 s delay period, while we used fixed windows not always aligned to the beta bursts. Still, the results for the trial-by-trial correlation analysis confirm their findings. Furthermore, we found no relationship between variability in spike count or beta amplitude and the strength of task-related or trial-by-trial correlation across pairs. Thus, the handful of beta-neuron pairs with a significant trial-by-trial correlation did not correspond to those with particularly high spike count or beta amplitude variability. This suggests that the near absence of significant trial-by-trial correlations was not simply caused by the pre-SC epoch having smaller individual signal variability compared with across all trial epochs.

To evaluate to which degree this task-related correlation with LFP beta amplitude depended on the involvement of each neuron in coding for the upcoming movement, we also analyzed the neuronal nonpreferred movement direction. The proportions of significantly correlated pairs were comparable for preferred and nonpreferred movement directions. However there were more significant negative correlations for the preferred direction compared with the nonpreferred. This difference did not reflect a change in correlation sign within individual pairs but was caused by nonoverlapping subsets of pairs with significant correlation for each movement direction. During movement preparation and execution, the neurons discharged less, per definition, in the nonpreferred compared with the preferred movement direction (Fig. 2). This could lead to larger proportions of neurons having a positive correlation with beta amplitude for their nonpreferred direction, as beta amplitude also decreased after the cue and during movement execution. It favors the interpretation that these beta-neuron correlations simply reflect to which degree the 2 signals are comodulated by the behavioral task.

Finally, we obtained very similar results for both beta bands in monkey M. Thus, no clear distinction can be made concerning potential functional roles of each band in this study, beyond the conclusion that there is no intrinsic relationship between oscillation amplitude and spike count of individual neurons for any of the 2 beta bands.

### No Need for Several Processes Underlying Motor Cortical Beta Amplitude Modulations

Rule et al. (2017) pointed out that beta amplitude decreases at movement onset, roughly when neurons in motor cortex are generally mostly active (see also Best et al. 2017; Khanna and Carmena 2017). This observation was in contradiction to the lack of a systematic relationship between beta amplitude and firing rate in their main analysis. They therefore proposed that 2 different processes govern motor cortical beta amplitude variability. One underlies the beta amplitude decrease around movement onset and is linked to large modulations in spiking rate. Another underlies the transient beta bursts during steady-state delays, lacking overt movements and decoupled from modulations in spiking activity.

Instead, we propose that there is no intrinsic relationship between LFP beta amplitude and neuronal firing rate. Thus, the significant task-related correlations observed in this study and the beta-to-rate mapping described in Canolty et al. (2012) are rather reflections of the beta amplitude (burst probability) and firing rate (spiking probability) both being modulated by the task events, however independently from each other. This conclusion is supported by our trial-shuffling control analysis. Thus, there is no need for different processes underlying modulations of beta bursts in steady-state situations compared with the suppression of beta bursts during movement execution (as well as after visual cues, see Kilavik et al. 2013; Zaepffel et al. 2013). Note that even if the underlying generating mechanisms might remain the same, this does not exclude potentially different functional roles for beta oscillation bursts occurring during cue anticipation, during movement preparation or postmovement (Kilavik et al. 2013; Torrecillos et al. 2015).

### Phase-Locking of Spikes to LFP Beta Oscillations

The lack of an intrinsic relationship between LFP beta amplitude and neuronal activation level (rate) does not exclude other relationships between beta oscillations and neuronal spiking activity. As we demonstrate in this dataset, confirming several previous studies (Murthy and Fetz 1996; Donoghue et al. 1998; Baker et al. 1999; Denker et al. 2011; Canolty et al. 2012; Engelhard et al. 2013; Riehle et al. 2018), there is significant locking of spike times to LFP beta oscillation phase for many neurons in motor cortex. This confirms that these oscillations are at least partly locally generated, including both bands in monkey M. Such phase locking may result in rhythmic synchronization among populations of neurons thereby increasing their concerted impact on postsynaptic targets without necessarily modifying their spike rates (Destexhe and Paré 1999; Azouz and Gray 2000).

## Conclusion

We conclude that there is no intrinsic relationship between the firing rate of individual neurons and LFP beta oscillation amplitude in macaque motor cortex, beyond each signal being concurrently modulated by external factors such as the behavioral task. As a final remark, understanding the mechanistic significance of beta oscillations as observed in the intracortical LFP is also highly relevant for studies in closely related fields using human participants. An extensive body of literature inquires the relationship between beta oscillations and task behavior, aiming at mechanistic understanding at the neuronal level using noninvasive techniques in the human. It is therefore crucial that we understand the relationship between these oscillations and the underlying spiking activity of individual neurons, across different levels of temporal precision, ranging from precise phase-locking to the herein addressed slower amplitude modulations.

## Notes

The authors wish to thank Adrian Ponce-Alvarez for participating in animal training and data recording; Ivan Balansard and Marc Martin for animal care; and Joel Baurberg, Alain De Moya, and Xavier Degiovanni for technical assistance. *Conflict of Interest*: None declared.

## Funding

Agence Nationale de la Recherche (grant numbers ANR-NEUR-05-045-1, ANR-18-CE37-0018-01); Ministère de l’Enseignement Supérieur et de la Recherche (PhD grant to J.C.).
